# Schutz der Rechte und Freiheiten von Personen bei der Datenverarbeitung im Gesundheitsbereich: Der Risikoansatz der EU-Datenschutz-Grundverordnung (DGSVO)

**DOI:** 10.1007/s00103-022-03652-6

**Published:** 2023-01-17

**Authors:** Fruzsina Molnár-Gábor

**Affiliations:** 1 Juristische Fakultät, Ruprecht-Karls-Universität Heidelberg, Heidelberg, Deutschland; 2 BioQuant Zentrum (BQ049), Im Neuenheimer Feld 267, 69120 Heidelberg, Deutschland

**Keywords:** Personenbezug, De-Identifizierung, Gesundheitsdatenverarbeitung, Risiken, Sichere Datenräume, Identifiability, De-identification, Health data processing, Risks, Secure data spaces

## Abstract

Die Zusammenführung von sensiblen Daten und die Rückführung ihrer Analyseergebnisse zu den betroffenen Personen ist ein wesentlicher Bestandteil der Datenverarbeitung im Gesundheitsbereich. Dies stellt eine besondere Herausforderung für den Datenschutz und damit für dessen wahren Zweck, den Schutz der Betroffenen, dar. Der Grund ist, dass die wissenschaftlichen und gesundheitlichen Erkenntnisse oft auf bestimmten Merkmalen in den Datensätzen fußen, die in ihrer Eigenschaft als personenbezogen beibehalten werden sollten, um die Ergebnisse der Datenanalyse fruchtbar werden zu lassen. Die Datenschutz-Grundverordnung (DSGVO) der Europäischen Union (EU) legt einen risikobasierten Ansatz fest, der sowohl die Frage des Personenbezugs von Daten als auch die Verhältnismäßigkeit ihrer Verarbeitung bestimmt.

In diesem Beitrag wird analysiert, wie der risikobasierte Ansatz den Anwendungsbereich der DSGVO eröffnet und mit den Risiken für die Rechte und Freiheiten der betroffenen Personen in Verbindung steht, die durch die Verarbeitung personenbezogener Daten entstehen. Darüber hinaus wird der Frage nachgegangen, inwieweit der risikobasierte Ansatz der DSGVO die Regeln für den internationalen Datentransfer beeinflusst, und erklärt, wie die internationale Datenverarbeitung im Gesundheitssektor auf seiner Grundlage zurzeit organisiert wird.

Insgesamt gibt die vorliegende Analyse Aufschluss darüber, wie die technischen Maßnahmen der Datenverarbeitung und die organisatorischen Maßnahmen zu deren Handhabung dazu beitragen können, die Verhältnismäßigkeit der Datenverarbeitung nach der DSGVO zu wahren, die im Wesentlichen als risikobasiert bestimmt werden kann, um zugleich der Spezifität der Datenverarbeitung im Gesundheitsbereich Rechnung zu tragen.

## Abstract (en)

Merging sensitive data and tracing their analysis results back to the data subjects is an essential part of data processing in the health sector. This challenges the protection of the data and thus its very purpose, the protection of the data subjects, since the scientific and health findings are often based on certain characteristics in the datasets, which should be preserved in their property as personal in order to make the results of the data analysis fruitful. The EU General Data Protection Regulation (GDPR) establishes a risk-based approach that determines both the identifiability of data and the proportionality of their processing.

This paper analyses how the risk-based approach opens the scope of the GDPR and relates it to the risks for the rights and freedoms of data subjects posed by the processing of personal data. Furthermore, the question is explored to what extent the risk-based approach of the GDPR influences the rules for international data transfer and how international data processing in the health sector is currently organised on its basis.

Overall, the present analysis sheds light on how the technical measures of data processing and the organisational measures for handling them can contribute to maintaining the proportionality of data processing under the GDPR, which can essentially be determined on a risk-based basis, while at the same time taking into account the specificity of data processing in the health sector.

## Hintergrund: Der Risikobegriff im Datenschutzrecht

Der Begriff des Risikos wird in der Alltagssprache als Synonym für „Bedrohung“ und „Unsicherheit“ verwendet.^1^ Auch im juristischen Sprachgebrauch ist die Unsicherheit ein bestimmendes Element des Risikobegriffs. Er wird in Bezug auf Szenarien verwendet, deren Eintrittswahrscheinlichkeit und Schadensmöglichkeit ungewiss sind.^2^

Der Begriff des Risikos wird in der Datenschutz-Grundverordnung (DSGVO)^3^ in den Erwägungsgründen 75 und 76 sowie im Erwägungsgrund (ErwG) 94 Satz 2 DSGVO erklärt. In ErwG 75 DSGVO werden Beispiele für mögliche Schäden aufgeführt, die durch die Verarbeitung personenbezogener Daten entstehen können.^4^ Dabei müssen physische, materielle und immaterielle Schäden berücksichtigt werden. In ErwG 76 DSGVO werden die Risikoelemente, die Eintrittswahrscheinlichkeit und die Schwere des Schadens definiert. Um die möglichen Risiken durch eine konkrete Datenverarbeitung präzise zu erfassen, werden die beiden Elemente auf der Grundlage von Art, Umfang, Umständen und Zwecken der Verarbeitung bestimmt. Darüber hinaus wird in ErwG 94 Satz 1 und Satz 2 DSGVO auf hohe Risiken hingewiesen, die von einer Datenverarbeitung ausgehen und von einfachen Risiken zu unterscheiden sind.

Aus diesen Erläuterungen leitet die Konferenz der Datenschutzaufsichtsbehörden die Definition des Risikos im Sinne der DSGVO ab, der zufolge es die Möglichkeit des Eintritts eines Ereignisses bedeutet, das selbst einen Schaden darstellt oder zu einem Schaden für natürliche Personen führen kann.^5^^,^^6^

Zielsetzung der Verordnung nach Art. 1 Abs. 1 DSGVO ist es, sowohl den Schutz der Betroffenenrechte als auch den freien Datenverkehr zu gewährleisten. Einschränkungen der Betroffenenrechte zugunsten der Datenverarbeitung dürfen nur unter den Bedingungen des Art. 8 Abs. 2 Grundrechtecharta (GRCh) und Art. 52 Abs. 1 GRCh vorgenommen werden (ErwG 4 Satz 2 DSGVO).^7^ Dementsprechend bedürfen Einschränkungen einer gesetzlichen Grundlage und müssen den Wesensgehalt des Rechts auf Datenschutz wahren. Außerdem müssen die getroffenen Maßnahmen verhältnismäßig sein.^8^ Die Verhältnismäßigkeit setzt eine Prüfung der Erforderlichkeit einer Maßnahme hinsichtlich der mit ihr verfolgten Zielverankerung voraus, ferner ihre Geeignetheit und Angemessenheit. Zwischen den verursachten Nachteilen und den verfolgten Zielen muss ein angemessenes Verhältnis bestehen. Bei der Bestimmung der Verhältnismäßigkeit müssen daher die Risiken für die Rechte und Freiheiten natürlicher Personen, die von einer bestimmten Datenverarbeitung betroffen sein können, berücksichtigt werden.^9^ Neben dem Recht auf Schutz personenbezogener Daten können auch alle Freiheits- und Grundrechte aus der GRCh^10^ sowie durch das Sekundärrecht garantierte Rechte betroffen sein^11^. In ErwG 75 führt die DSGVO beispielhafte Risikoszenarien auf, die auf Rechte und Freiheiten hinweisen, die durch die Verarbeitung beeinträchtigt werden können.^12^

Um einer Beeinträchtigung der Rechte und Freiheiten der Betroffenen durch die Datenverarbeitung entgegenzuwirken und mögliche Schäden zu vermeiden, bedarf es bei der Verarbeitung von personenbezogenen Daten Mechanismen, die eine informierte und rationale Entscheidungsfindung im Hinblick auf ihre Risiken ermöglichen. Im Mittelpunkt steht dabei die Frage, welche rechtlichen Anforderungen an Risikoprognosen gestellt werden müssen, um die Folgen verschiedener Handlungsoptionen bei der Datenverarbeitung und verschiedener Verarbeitungsschritte bewerten und vergleichen zu können. Darüber hinaus ist zu definieren, welche Mechanismen geeignet sind, den Prozess der Datenverarbeitung im Hinblick auf das mit der Verarbeitung verbundene Risiko innerhalb des rechtlich definierten Rahmens zu halten. Dabei kann zwischen einer inhaltlichen Bestimmung relevanter Entscheidungen und einer Steuerung durch Prozeduralisierung^13^, etwa der organisatorischen Gestaltung der Entscheidungsfindung, unterschieden werden. Darüber hinaus ist die Frage von Bedeutung, wie Verantwortung für die Datenverarbeitung als Risiko bzw. für eingetretene Schäden bestimmt werden kann.

Je nach dem Risiko der Datenverarbeitung legt die DSGVO inhaltliche und prozedurale Vorschriften fest. Darüber hinaus spielt das Risiko auch in Bestimmungen der DSGVO eine Rolle, die den Begriff des Risikos nicht ausdrücklich erwähnen, aber zumindest indirekt auf eines seiner Elemente, die Wahrscheinlichkeit des Eintretens einer Beeinträchtigung des Betroffenen, verweisen.

## Der Risikoansatz der Datenschutz-Grundverordnung (DSGVO)

### Die Rolle des Risikos bei der Eröffnung des sachlichen Anwendungsbereichs der DSGVO: Der Personenbezug von Daten nach der DSGVO

Der sachliche Anwendungsbereich der DSGVO ist für die Verarbeitung personenbezogener Daten gem. Art. 2 Abs. 1 DSGVO eröffnet. Art. 4 Nr. 1 DSGVO definiert personenbezogene Daten als alle Informationen, die sich auf eine identifizierte oder identifizierbare natürliche Person beziehen. Wenn die Daten nicht einer Person zugeordnet werden können, ist deren Identifizierung weder mittelbar noch unmittelbar möglich. Es liegen keine personenbezogenen Daten vor, sondern, herkömmlich gesprochen, anonyme Daten.

Nach ErwG 26 Satz 3 DSGVO hängt die Identifizierbarkeit davon ab, ob der Bezug zu einer Person durch den für die Verarbeitung Verantwortlichen oder durch eine andere Person hergestellt werden kann. Dabei sind alle Mittel zu berücksichtigen, die nach allgemeinem Ermessen wahrscheinlich zur Identifizierung der Person verwendet werden können. Bei der Feststellung, ob die Mittel zur Identifizierung der Person nach allgemeinem Ermessen wahrscheinlich genutzt werden, sind nach ErwG 26 Satz 4 DSGVO alle objektiven Faktoren zu berücksichtigen. Dazu gehören etwa die Kosten der Identifizierung und der dafür erforderliche Zeitaufwand. Auch der Stand der Technik spielt eine wichtige Rolle.

Nach Ansicht des Europäischen Datenschutzausschusses muss jede Anonymisierung vollständig unumkehrbar sein.^14^ Darüber hinaus wird vertreten, dass die Anonymisierung „zukunftssicher“ sein muss, was bedeutet, dass sie unempfindlich gegenüber neuen Technologien sein sollte.^15^ Neben der Generalisierung und Randomisierung wird die Entfernung von Merkmalen als ideale technische Maßnahme zur Anonymisierung vorgestellt.^16^

Die DSGVO verwendet den Begriff Anonymisierung nicht und spricht lediglich vom Personenbezug der Daten. Dieser hängt von den oben genannten Faktoren, wie dem Stand der Technik, den Verarbeitern, Datenverknüpfungen und damit insgesamt von den Modalitäten der konkreten Datenverarbeitung, ab.^17^ Diese bilden in ihrer Gesamtheit den Verarbeitungskontext^18^^,^^19^, dessen Beurteilung Aufschluss über die Wahrscheinlichkeit der Identifizierung geben kann. Der Personenbezug von Daten kann je nach Verarbeitungskontext variieren oder gar hergestellt werden, ist damit aber in jedem Fall abgestuft. Demgegenüber vermittelt der Begriff der Anonymität eine Absolutheit, eine Binarität des Personenbezugs, die deren Kontextabhängigkeit nicht berücksichtigt.^20^

Die Verarbeitungsziele im Gesundheitskontext können regelmäßig nur erreicht werden, wenn zumindest eine indirekte Rückverfolgbarkeit der Daten zu den betroffenen Patienten und Probanden gegeben ist.^21^

Die Ätiologie von Krankheiten und das Verständnis der Rolle der verschiedenen Gesundheitsfaktoren bei der Krankheitsentwicklung erfordern die Verarbeitung personenbezogener Daten. Die Löschung oder Entfernung bestimmter Variablen aus den Gesundheitsdaten zum Zwecke der Anonymisierung steht in direktem Widerspruch zu dem Grund, aus dem die Verarbeitung regelmäßig erfolgt, da eine solche Änderung die Aussagekraft der Daten beeinträchtigen würde. Ein Beispiel hierfür ist die Entfernung von Metadaten eines bestimmten Formats^22^ aus Krebsbildgebungsdatensätzen, bei denen es sich um indirekte Identifikatoren wie die Seriennummer des Geräteherstellers handeln kann, deren Verlust jedoch die Rückverfolgbarkeit zum Patienten beeinträchtigen kann.

Der Verlust der Rückverfolgbarkeit kann besonders schwerwiegende Folgen bei klinischen Studien haben, wo es nicht nur von entscheidender Bedeutung ist, sondern oft auch zur Pflicht wird, die Studienergebnisse in identifizierbarer Form zu verifizieren und den Patienten zur Verfügung zu stellen.^23^ Darüber hinaus können Methoden der Datenanonymisierung zum systematischen Ausschluss von Angehörigen kleiner Bevölkerungsgruppen von der Aufnahme in wissenschaftliche Datensätze führen, da indirekte Identifikatoren, die in Datensätzen seltener vorkommen, mit größerer Wahrscheinlichkeit den De-Identifizierungsmethoden zum Opfer fallen.^24^

Anonymisierungstechniken können, wenn sie nach unterschiedlichen Spezifikationen und auf der Grundlage eines unterschiedlichen Verständnisses von personenbezogenen Daten umgesetzt werden, zu Unterschieden in der Datenqualität und damit zu einer geringeren technischen Interoperabilität zwischen Datensätzen führen.^25^ Dies kann die Reproduzierbarkeit und Vergleichbarkeit von Forschungs- und Versorgungsergebnissen beeinträchtigen und die statistische Validität der Ergebnisse aufweichen.^26^ Insgesamt wäre es möglich, dass dadurch die Anonymisierung im Gesundheitsbereich nicht genutzt werden kann, ohne das Potenzial von Forschung und Versorgung drastisch zu schmälern.

Die Pseudonymisierung nach Art. 4 Nr. 5 DSGVO beschreibt eine Verarbeitung nach den kumulativen Kriterien, dass (1) die personenbezogenen Daten ohne Hinzuziehung zusätzlicher Informationen nicht mehr einer spezifischen betroffenen Person zugeordnet werden können, sofern (2) diese zusätzlichen Informationen gesondert aufbewahrt werden und (3) technischen und organisatorischen Maßnahmen unterliegen, die gewährleisten, dass die personenbezogenen Daten nicht einer natürlichen Person zugewiesen werden. Pseudonymisierte Daten stellen einen Unterfall von personenbezogenen Daten dar, ErwG 26 DSGVO.^27^ Je nach Relevanz des Zusatzwissens Dritter, die keinen Zugang zum Pseudonymisierungsschlüssel haben, wird vertreten, dass die Daten für diese Dritten keinen Personenbezug aufweisen.^28^ Da die Verarbeitungskontexte in der Medizin veränderlich sind, ist fraglich, ob diese Sichtweise über den gesamten Datenlebenszyklus Bestand haben kann. In Bezug auf den Verantwortlichen lässt sich festhalten, dass auch, wenn die Pseudonyme von einem vertrauenswürdigen Dritten vergeben werden^29^, der Verantwortliche in der Regel weiterhin fähig sein muss, die Daten auf die Patienten zurückzuführen. Des Weiteren ist zu beachten, dass Identifikationsnummern häufig als Metadaten bekannt gegeben werden, um die darunterliegenden Datensätze beim jeweiligen Verantwortlichen auffindbar zu machen. Die Veröffentlichung solcher personenbezogener Identifikatoren als Metadaten durch den Verantwortlichen kann bspw. im Anwendungsbereich landesrechtlicher Datenschutzvorschriften dem strengen Einwilligungserfordernis unterliegen.^30^ Dennoch bietet die Pseudonymisierung einen sehr starken Schutz und kann bewirken, dass in der Abwägung zwischen den Verarbeiterinteressen und den Schutzinteressen die Risiken für die Betroffenen erheblich gesenkt werden (ErwG 28 DSGVO) und die Einhaltung der Datenschutzpflichten und damit die Verarbeitung insgesamt vereinfacht wird^31^, denn es kann auf besondere (weitere) Schutzmaßnahmen verzichtet werden^32^.

### Personenbezug als Risiko

Bei der Frage, ob durch verschiedene Maßnahmen die Herstellung des Personenbezugs von Daten verhindert werden kann, wird in der Literatur^33^ und der Rechtsprechung auf die „anonymisierende Wirkung“ von Maßnahmen hingewiesen.^34^ Auch technische Maßnahmen, die die Daten verändern, unterbrechen zwar den unmittelbaren Personenbezug in den meisten Verarbeitungskontexten, können einen mittelbaren Personenbezug jedoch nicht vollständig ausschließen, da der konkrete Verarbeitungskontext über den Erfolg der De-Identifizierung mitentscheidet und die Stärke der technischen De-Identifizierung (mit)bestimmt.^35^ Wenn der Akteur, der die Daten de-identifiziert, auch den Rohdatensatz, andere identifizierende Daten und/oder den Schlüssel kontrolliert, mit dem er den Prozess der De-Identifizierung rückgängig machen könnte, ist eine Re-Identifizierung der Daten leicht möglich. Für einen Dritten, der die Daten erhält, kann die Re-Identifizierung der betroffenen Person auf ihrer Grundlage jedoch sehr viel schwieriger und unpraktisch sein.^36^ Wenn Daten nur in einer streng kontrollierten und geschützten Umgebung verarbeitet werden und nicht heruntergeladen werden können, ist die Möglichkeit, sie mit anderen Daten zu verknüpfen, viel geringer, als wenn dieselben Daten öffentlich zugänglich sind.^37^ Darüber hinaus sind Gesundheitsdaten und insbesondere genetische Daten durch weitere Datenverknüpfungen in hohem Maße identifizierend.^38^

Insgesamt ist entscheidend, wie wahrscheinlich die Herstellung eines Personenbezugs der Daten ist. Es sind nur solche Mittel zu berücksichtigen, die „vernünftigerweise“ zur Herstellung des Personenbezugs eingesetzt werden können.^39^ Die Einstufung von Daten als „anonym“ oder als „nicht personenbezogen“ hängt also von der Intensität des Aufwands ab, der erforderlich ist, um einen Personenbezug herzustellen.^40^^,^^41^ Die Bewertung der Intensität hängt wiederum von der Vernünftigkeitsschwelle ab: Der Aufwand zur Herstellung des Personenbezugs darf nicht unverhältnismäßig sein. Auch technische Maßnahmen, die die Herstellung des Personenbezugs ermöglichen, wie das in ErwG 26 Satz 3 DSGVO genannte „Aussondern“ („singling out“), müssen vernünftigerweise einsetzbar sein. Die bloße Tatsache, dass es Maßnahmen gibt, die die Herstellung des Personenbezugs ermöglichen, bedeutet nicht, dass sie mit verhältnismäßigem Aufwand eingesetzt werden können.^42^

Die Bewertung der Wahrscheinlichkeit kann mithilfe einer Risikovorhersage erfolgen, wobei sowohl die dem potenziellen Datenverarbeiter selbst innewohnenden Faktoren, wie sein Wissen, als auch die von den Datenverarbeitern einsetzbaren Mittel der Zuordnung berücksichtigt werden.^43^ In der Praxis geschieht dies in der Regel dadurch, dass ein maximal großes Risiko angenommen wird und die Bewertung der Eintrittswahrscheinlichkeit anhand objektiver Faktoren erfolgt, die auf der Grundlage eines allgemeinen Ermessens angewandt werden können.

### Risiko für die Rechte und Freiheiten der Betroffenen

Der Risikoansatz spielt auch bei der Beurteilung der möglichen Beeinträchtigung der Betroffenenrechte durch die Verarbeitung personenbezogener Daten eine Rolle. Die Maßnahmen, die zu ergreifen sind, um möglichen Beeinträchtigungen entgegenzuwirken, sind auf der Grundlage einer Abwägung zu bestimmen, bei der die Interessen der Betroffenen mit den Interessen der Datenverarbeiter in einen Ausgleich zu bringen sind.^44^^,^^45^

Diese Abwägung wird zunächst durch die Tatsache beeinflusst, dass die DSGVO zahlreiche sektorspezifische Interessen privilegiert.^46^ Die daraus resultierende Risikobewertung spiegelt sich in der Bestimmung der Rechtsgrundlage für die Datenverarbeitung nach Art. 6 Abs. 1 DSGVO wider.^47^ Darüber hinaus schreibt die Kompatibilitätsprüfung im Zuge der Weiterverarbeitung gem. Art. 6 Abs. 4 DSGVO die Berücksichtigung der Folgen für die Betroffenen vor.^48^ Die Abwägung der Risiken für die Betroffenen unter Berücksichtigung bestimmter Verarbeiterinteressen zeigt sich auch in den Ausnahmen vom Verbot der Verarbeitung besonderer Kategorien personenbezogener Daten gem. Art. 9 Abs. 2 DSGVO.^49^^,^^50^ Die zusätzliche Heranziehung eines Ausnahmetatbestands berücksichtigt die Sensibilität der Daten, einschließlich der Gesundheitsdaten, da deren Verarbeitung zu einem höheren Risiko für die Betroffenen führen kann. Gleichzeitig werden die Ausnahmen vom Verbot der Verarbeitung sensibler Daten nur für bestimmte Verarbeitungen geschaffen, die bestimmte Zwecke verfolgen, einschließlich der wissenschaftlichen Forschung und der Gesundheitsversorgung.^51^

Darüber hinaus ist der Risikobegriff bei den Verarbeiterpflichten, bei der datenschutzfreundlichen Technikgestaltung und bei der Implementierung von technisch-organisatorischen Maßnahmen und den damit einhergehenden Fragen der Datensicherheit von Bedeutung.

Der Risikoansatz soll es ermöglichen, den Umfang der Pflichten von Datenverarbeitern an das jeweilige Risiko ihrer Verarbeitung anzupassen.^52^ Einerseits kann die Risikoanalyse bereits einen Einfluss auf das Bestehen bestimmter Pflichten haben.^53^ Andererseits bestimmt die Risikobewertung auch die Art und Weise, wie die Pflichten erfüllt werden.^54^ So sind nach Art. 24 Abs. 1 DSGVO bei der Risikobewertung vor der Datenverarbeitung die Art der Verarbeitung, ihr Umfang, ihre Umstände und ihr Zweck sowie die Eintrittswahrscheinlichkeit und Schwere der Risiken für die Rechte und Freiheiten natürlicher Personen zu berücksichtigen.^55^ Es ist zu betonen, dass diese Risikobewertung den für die Verarbeitung Verantwortlichen nicht von seinen Pflichten entbindet, sondern lediglich deren risikoadäquate Anpassung rechtfertigt.^56^ Führt die Einschätzung zu dem Ergebnis, dass die Verarbeitung mit hohen Risiken verbunden ist, muss eine Datenschutz-Folgenabschätzung nach Art. 35 DSGVO durchgeführt werden. Im Rahmen der Verpflichtung zur datenschutzfreundlichen Technikgestaltung nach Art. 25 Abs. 1 DSGVO wird eine Risikobewertung eigens vorgeschrieben.^57^

Art. 32 Abs. 1 DSGVO verpflichtet Verantwortliche und Auftragsverarbeiter, technische und organisatorische Maßnahmen zu treffen, die ein dem Risiko angemessenes Datenschutzniveau gewährleisten. Das Risiko wird anhand der Art und Weise der Verarbeitung, des Verarbeitungskontexts und der Intensität des drohenden Schadens für die Betroffenen sowie seiner Eintrittswahrscheinlichkeit bestimmt.^58^^,^^59^ Dabei sind das technisch Machbare und die wirtschaftlichen Faktoren bei der Festlegung geeigneter Maßnahmen zur Eindämmung des Risikos in die Überlegungen einzubeziehen.^60^^,^^61^

Art. 32 Abs. 2 DSGVO listet einen Katalog von Ereignissen auf, die eine Störung darstellen (bspw. Vernichtung, Verlust, im Sinne der Verletzung des Schutzes der personenbezogenen Daten), und knüpft damit das Schutzniveau an die Schutzbedürftigkeit der personenbezogenen Daten.^62^ In der Praxis muss der jeweilige Schutzbedarf der verschiedenen personenbezogenen Daten anhand von typischen Schadenszenarien ermittelt werden, bevor dann die Einstufung in Schutzbedarfskategorien erfolgt.^63^ Auf Grundlage der Schutzbedarfskategorien werden geeignete technische und organisatorische Maßnahmen identifiziert und umgesetzt.^64^ Zum Nachweis der Einhaltung der Anforderungen des Art. 32 Abs. 1 DSGVO können anerkannte Zertifizierungsverfahren und Verhaltenskodizes verwendet werden, die kontextspezifische Eigenschaften der Datenverarbeitung abbilden können.^65^

### Der Einfluss des Risikoansatzes auf internationale Datentransfers

#### Verhältnismäßigkeit der Datenverarbeitung nach der DSGVO und Vorschriften für Datentransfers

Für internationale Datenübermittlungen sieht Kapitel V der DSGVO eine zweistufige Prüfung vor. In der ersten Stufe wird untersucht, ob die Daten rechtmäßig verarbeitet werden, um in der zweiten Stufe der Frage nachzugehen, ob die gewählten Datenübermittlungsmechanismen mit den Bestimmungen von Kapitel V der DSGVO übereinstimmen.^66^ Die Vorschriften der Art. 44 ff. DSGVO sehen vor, dass die Datenübermittlungsvorschriften des Kapitels V der DSGVO so anzuwenden sind, dass das in der Verordnung vorgesehene Schutzniveau nicht unterlaufen wird. Dies ist gewährleistet, wenn das Datenschutzniveau im Drittland „angemessen“ ist.

Angemessen ist das Schutzniveau im Drittland, wenn es aufgrund dessen innerstaatlichen Rechtsvorschriften und internationalen Verpflichtungen tatsächlich dem in der EU garantierten Niveau der Sache nach gleichwertig ist.^67^ Dementsprechend ist ein identisches Schutzniveau nicht erforderlich, aber das Schutzniveau muss dem Unionsrecht funktional nahekommen.^68^^,^^69^ Die Bewertung der Angemessenheit erfolgt damit anhand des Datenschutzniveaus des Drittlandes im Vergleich zu dem der GRCh und ihrer sekundärrechtlichen Konkretisierung durch die DSGVO.

Die Gestaltung der Übermittlung richtet sich damit auch nach geeigneten Garantien für die Rechte der betroffenen Personen, die am Grundsatz der Verhältnismäßigkeit zu messen sind.^70^^,^^71^ Dies wird entweder durch eine Entscheidung der Europäischen Kommission für ein Drittland oder einen Sektor in einem Drittland festgelegt oder durch die Verwendung von vordefinierten Übermittlungsmechanismen sichergestellt.^72^ Im letzteren Fall, wenn die Bewertung des Datenschutzniveaus in dem Drittland ergibt, dass die dortigen Rechtsvorschriften und/oder Praktiken die Wirksamkeit der vom Datenexporteur im Rahmen von Art. 46 DSGVO gewählten Übermittlungsmechanismen beeinträchtigen und damit die Angemessenheit der Datenverarbeitung in dem Drittland in Frage stellen, müssen zusätzliche Maßnahmen ergriffen werden.^73^ Die zusätzlichen Maßnahmen dienen dem Zweck, die fehlende Angemessenheit des Datenschutzniveaus im Drittland auszugleichen.^74^ Durch diese kompensatorische Wirkung können sie die Verhältnismäßigkeit der Datenverarbeitung sicherstellen, indem sie die Risiken für die Rechte und Freiheiten der betroffenen Personen minimieren.

Das Hauptziel der DSGVO besteht darin, einen Rahmen zu schaffen, in dem der für die Verarbeitung Verantwortliche die Risiken der Verarbeitung personenbezogener Daten und den Schutz der Interessen des Betroffenen mit den Verarbeiterinteressen abwägt. Die Mechanismen der DSGVO für internationale Datenübermittlungen sollen sicherstellen, dass die nach dem EU-Datenschutzrecht geltenden Vorgaben auch nach der Übermittlung von Daten außerhalb der EU und des Europäischen Wirtschaftsraums (EWR) wirksam sind, einschließlich der verhältnismäßigen Abwägung von Nutzen und Schaden durch die Verarbeitung. Da lediglich vierzehn Länder einen Angemessenheitsbeschluss der Europäischen Kommission erhalten haben^75^ und es für die Datenexporteure sehr umständlich ist, die Rechtslage in Drittländern aus der Perspektive der Angemessenheit einzuschätzen^76^, sind im Gesundheitsbereich neue Lösungen notwendig, um eine Datenverarbeitung im Einklang mit den anzuwendenden datenschutzrechtlichen Bestimmungen zu gewährleisten.

#### Lösungsansätze

##### Datenschutzrechtliche Rollenverteilung.

**Figure 1:**
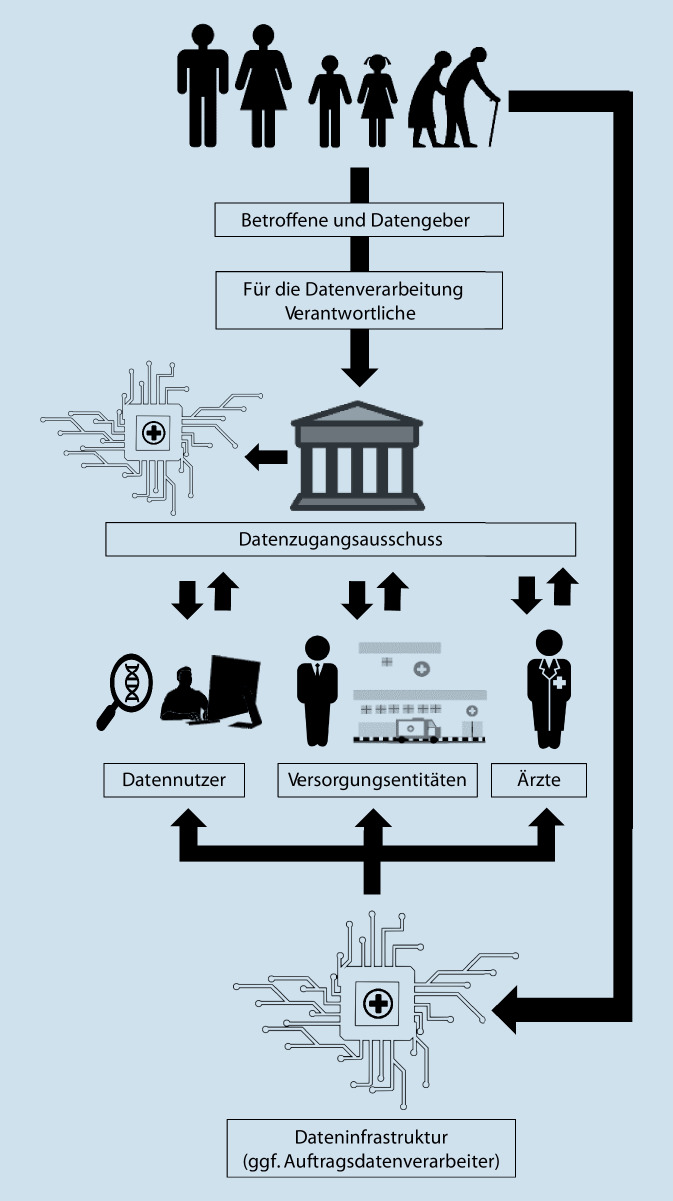

Die DSGVO sieht verschiedene Rollen für Datenverarbeiter vor, z. B. die des für die Verarbeitung Verantwortlichen, die des Auftragsverarbeiters oder die des gemeinsam Verantwortlichen. In Abb. 1 ist eine mögliche vereinfachte Struktur der wesentlichen Rollenverteilung dargestellt und in einen erweiterten Kontext der medizinischen Datenverarbeitung gestellt. Ein für die Verarbeitung Verantwortlicher bestimmt die Zwecke und wesentlichen Mittel der Verarbeitung (Art. 4 Nr. 7 DSGVO).^77^ Gemeinsam für die Verarbeitung Verantwortliche werden eingesetzt, wenn eines der beiden Elemente gemeinsam festgelegt wird (Art. 26 Abs. 1 Satz 1 DSGVO).^78^^,^^79^^,^^80^ Auftragsverarbeiter führen die Datenverarbeitungsschritte nur nach den Anweisungen des für die Verarbeitung Verantwortlichen durch (Art. 4 Nr. 8 DSGVO). Die Pflichten unterscheiden sich je nach Rolle, wobei die Zuweisung von Rollen die Einhaltung der Anforderungen an eine verhältnismäßige Datenverarbeitung erheblich erleichtern kann.

Da die Datenverarbeiter im Gesundheitswesen aufgrund kleinteiliger Arbeitsabläufe und verteilter Kompetenzen die Zwecke und wesentlichen Methoden der Datenverarbeitung häufig gemeinsam festlegen, kann es bei Forschungskonsortien oder Kooperationen zwischen versorgungsrelevanten Akteuren leicht zu einer gesamtschuldnerischen Haftung nach Art. 26 Abs. 3 DSGVO kommen, die durch eine differenzierte Zuordnung von Pflichten und damit von Haftungsbereichen im Innenverhältnis geregelt werden muss.^81^^,^^82^^,^^83^

Darüber hinaus ist es oft schwierig, die wesentlichen von den nicht wesentlichen Mitteln zur Verarbeitung von Gesundheitsdaten zu trennen.^84^ Technische Lösungen für den Austausch von Gesundheitsdaten erfordern regelmäßig die Einschaltung eines technisch versierten Auftragsverarbeiters, dessen Tätigkeiten leicht einen wesentlichen Teil des Datenaustauschs darstellen können. Datenplattformen, die zunächst als Auftragsverarbeiter eine sichere Datenspeicherung anbieten, werden sich leicht in der Rolle des für die Verarbeitung Verantwortlichen befinden, wenn sie beispielsweise zur Erstellung von recherchierbaren Metadatenbibliotheken für personenbezogene Gesundheitsdaten beitragen.^85^ Des Weiteren nutzen Verantwortliche verschiedene Arten von Expertengremien, um ihre Arbeit zu unterstützen. Zu diesen Gremien gehören spezialisierte Ausschüsse wie z. B. Datenzugangsausschüsse, die für die Verwaltung des Zugangs zu sensiblen Datensätzen gemäß den Kriterien, die von den für die Verarbeitung Verantwortlichen festgelegt wurden, zuständig sind.^86^ Dabei muss die Bewertung potenzieller Risiken anhand vordefinierter Maßstäbe von der faktischen Festlegung von Maßstäben für den Datenzugang unterschieden werden, um zu vermeiden, dass diese Gremien eine datenschutzrechtliche Rolle, wie die des für die Verarbeitung Verantwortlichen, erhalten.

##### Datenföderation: Vor- und Nachteile.

**Figure 2:**
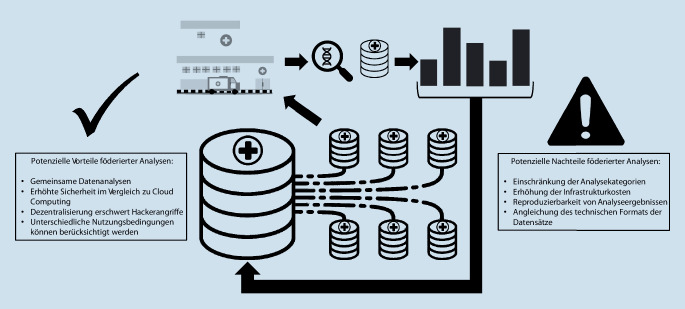

Föderierte Technologien ermöglichen die gemeinsame Analyse dezentral gehaltener Datensätze.^87^ In Abb. 2 werden Vor- und Nachteile der föderierten Datenanalyse gezeigt. So können beispielsweise verschiedene Gesundheitsdienstleister gemeinsam statistische Analysen durchführen und maschinelle Lernmodelle entwickeln, ohne die zugrundeliegenden Datensätze untereinander auszutauschen. Es werden lediglich aggregierte Ergebnisse oder Modellaktualisierungen übermittelt. Auf diese Weise kann jeder Gesundheitsdienstleister seine eigenen Spezifikationen für die Verarbeitung seiner Daten festlegen und die Kontrolle über den Zugriff behalten.^88^

Der Rückgriff auf föderierte Datenanalysetechniken kann jedoch technologisch aufwendig sein und die durchführbaren Analysekategorien stark einschränken. Die föderierte Analyse erfordert häufig eine Verdoppelung der technologischen Infrastruktur an jedem beteiligten Knotenpunkt, was die Infrastrukturkosten erhöht.^89^ Darüber hinaus kann es Schwierigkeiten bei der Gewährleistung der Reproduzierbarkeit von Analyseergebnissen und bei der Angleichung des technischen Formats verschiedener Datensätze geben, da die Föderationspartner die betreffenden Datensätze selbst nicht analysieren oder verändern können.^90^ Obwohl die Daten auf Patientenebene nicht zwischen den teilnehmenden Einrichtungen übertragen werden, hat sich gezeigt, dass die ausgetauschten Daten unter bestimmten Umständen sensible persönliche Informationen preisgeben können, was zur Herstellung des Personenbezugs durch Ableitung von Zugehörigkeiten zu einer Merkmalsgruppe oder einer Rekonstruktion von Merkmalen führt.^91^ Im Falle eines bedingten Personenbezugs kann dies letztlich auch den Anwendungsbereich der DSGVO für bestimmte Verarbeitungsschritte eröffnen, die als außerhalb des Anwendungsbereichs liegend vermutet wurden. Daraus kann sich die Notwendigkeit organisatorischer Maßnahmen ergeben, die den Verarbeitungskontext beeinflussen, um die Verhältnismäßigkeit auf der Grundlage der vorgenommenen Risikobewertung zu wahren.

##### Sichere Datenräume.

Vorhaben, die die Datenverarbeitung auf eine technische Infrastruktur innerhalb der EU beschränken, die es den Nutzern nicht ermöglicht, die betreffenden Daten herunterzuladen, anderweitig zu vervielfältigen oder zu modifizieren, senken das Risiko der Herstellung des Personenbezugs erheblich. Zwar könnte die Datenanalyse weiterhin in den Anwendungsbereich der DSGVO fallen, aber das Risiko für eine Beeinträchtigung der Rechte und Freiheiten der Betroffenen würde sehr niedrig ausfallen und in der Abwägung würden die widerstreitenden Verarbeiterinteressen in der Regel nicht überwiegen. Eine zusätzliche Pflicht zur Anonymisierung der Daten erscheint in einem solchen Verarbeitungskontext im Gesundheitsbereich für den Erkenntnisgewinn nicht sinnvoll. Angesichts der Beeinträchtigung der Verarbeiterinteressen ist es auch fraglich, ob dies einer verhältnismäßigen Abwägung entspricht.^92^

Des Weiteren wäre es überlegenswert, den Zugang zu Gesundheitsdaten in einem sicheren Datenraum nicht als internationalen Datentransfer zu qualifizieren, wenn Akteure außerhalb der EU/des EWR den Zugang begehren. Da der Zugriff auf Daten innerhalb einer EU-Infrastruktur nach technischen Spezifikationen erfolgen würde, die den Verhältnismäßigkeitsgrundsatz des EU-Datenschutzrechts beachten, wäre es nicht logisch, solche Datenverarbeitungstätigkeiten als Datenübermittlung zu behandeln.^93^ Wenn der Zugang zu den im Datenraum gehosteten Daten sowohl in der EU als auch außerhalb der EU unter den gleichen Bedingungen erfolgen würde, bestünde in der Tat keine Möglichkeit, dass solche Datenverarbeitungstätigkeiten die Datenschutzgarantien für die betroffenen Personen in der EU beeinträchtigen.^94^ Wenn durch technische Maßnahmen und organisatorische Anforderungen ein sicherer Datenraum geschaffen wird, der die kontinuierliche Anwendung der EU-Datenschutzstandards gewährleistet, liegt es auf der Hand, dass die auf einer solchen sicheren Plattform durchgeführte Datenverarbeitung nicht die Anwendung der DSGVO-Transfermechanismen auslösen würde.^95^

Eine weitere Verringerung des Risikos der Herstellung des Personenbezugs wäre denkbar, wenn auch die Datenanalyse durch den Datenanbieter im sicheren Raum durchgeführt würde. In diesem Fall würden Nutzer nur ihre Analysefragen einsenden, hätten aber keinen Zugang zu den Daten, auch nicht im sicheren Datenraum. Hierbei sind verfügbare Beschreibungen der im Datenraum gehaltenen Daten wichtig, um sie auffindbar zu machen.

Ein aktuell entstehender Datenraum ist der Europäische Gesundheitsdatenraum (European Health Data Space – EHDS). Der Verordnungsentwurf sieht ein Konzept vor, das einen grenzüberschreitenden Datenaustausch bzw. -zugang mittels getrennter Infrastrukturen – je nach den Verarbeitungszwecken der Gesundheitsversorgung und der Sekundärnutzung von Daten – ermöglicht.^96^^,^^97^ Der Verordnungsentwurf sieht umfangreiche Maßnahmen vor, mit denen die Verfügbarkeit von Daten für natürliche Personen und sonstige Datennutzer erheblich ausgebaut werden soll. Dies umfasst insbesondere auch die Einführung bzw. Benennung nationaler öffentlicher Stellen, denen umfangreiche Aufgaben zugewiesen werden.

Zum Schutz der Rechte natürlicher Personen und zur Wahrung der Grundsätze der Datenminimierung und Zweckbegrenzung sind elektronische Gesundheitsdaten grundsätzlich in anonymisierter Form für die Sekundärverwendung in der Forschung bereitzustellen, soweit dies zur Erreichung des Verarbeitungszwecks des Datennutzers genügt (Art. 44 Abs. 2 EHDS-Verordnungsentwurf (EHDS-VO-E)). Ist dies nicht der Fall, kann ein Zugang zu den Daten in pseudonymisierter Form erfolgen; Datennutzern ist es jedoch strikt untersagt, diese Daten zur (Re‑)Identifizierung heranzuziehen (Art. 44 Abs. 3 EHDS-VO-E). Zudem soll nur zu solchen Daten Zugang gewährt werden, die für den Verarbeitungszweck des Datennutzers relevant sind (Art. 44 Abs. 1 EHDS-VO-E).

Angesichts der Tatsache, dass die Datenverarbeitung auf die technische Infrastruktur des EHDS beschränkt werden soll, die es den Nutzern nicht ermöglicht, die betreffenden Daten herunterzuladen oder anderweitig zu vervielfältigen, scheint die anonymisierte Zurverfügungstellung von Daten nicht zu mehr Datenschutz und zudem zu einem Verlust des Analysewertes der Daten für die Forschung zu führen. Zugleich ist nicht nachvollziehbar, warum im EHDS nicht unterschieden wird zwischen der Bewertung des Datenschutzstandards für internationale wissenschaftliche Kooperationen, die auf der Verarbeitung von anonymen Daten beruhen, und solchen, die pseudonymisierte Daten heranziehen.^98^

## Zusammenfassende Bewertung des datenschutzrechtlichen Risikobegriffs und Konsequenzen für die Verarbeitung von Gesundheitsdaten

Das Risiko für die Rechte und Freiheiten der betroffenen Person bestimmt die Art und Weise der Datenverarbeitung. Dabei sind das Risiko der (Wieder‑)Herstellung eines Personenbezugs und das Risiko der Verarbeitung für die Rechte und Freiheiten der Betroffenen als voneinander abhängige Prognosen anzusehen, die in eine einheitliche Folgenabschätzung der Datenverarbeitung zusammengefasst werden können.^99^ Mit der zunehmenden Etablierung eines bedingten Personenbezugs wird deutlich, dass dieser nur in den seltensten Fällen absolut und kontextunabhängig verlangt werden kann.

Die Verringerung der Reichhaltigkeit des Datenmaterials durch Anonymisierungstechniken mindert allerdings den wissenschaftlichen und gesundheitsbezogenen Wert der Daten und schränkt das Potenzial zur Beantwortung von Fragen im Kontext von Forschung und Versorgung, der anwendbaren Forschungs- und Diagnosemethoden und der Relevanz der Forschungsergebnisse ein. Die unterschiedlichen rechtlichen Interpretationen der Anonymität beeinflussen die Bewertung der technischen Umsetzung der De-Identifizierung und können dadurch die Interoperabilität zwischen Datensätzen beeinträchtigen. Zu beachten ist, dass die De-Identifizierung und die kontextbezogene Anonymisierung immer neben technischen auch organisatorische Maßnahmen des Datenschutzes erfordern, auch wenn die Grenze zwischen technischen und organisatorischen Maßnahmen fließend ist.

Darüber hinaus wird die Dringlichkeit sektorspezifischer Datenschutzmaßnahmen deutlich, die sowohl die Frage des personenbezogenen Charakters der Verarbeitung klären als auch die Verhältnismäßigkeit der Verarbeitung kontextbasiert definieren können. Des Weiteren können kontextbezogene Verarbeitungsregeln auch dazu beitragen, die Übergänge zwischen datenschutzrechtlich relevanter und nicht relevanter Verarbeitung in einem bestimmten Bereich zu definieren, indem sie den Datenschutz immer in Bezug auf den typisierten Verarbeitungsvorgang definieren. Sie können Vorgaben etablieren, wie die betroffenen Daten verarbeitet werden dürfen, um ihre kontextbezogene Anonymität zu wahren, die allerdings gemessen an der Vernünftigkeitsschwelle einer echten Anonymität nur im jeweiligen Verarbeitungskontext entsprechen muss. Diese Maßnahmen können bspw. spezifische Zweckbestimmungen für die Verarbeitung, Datensicherheitsmaßnahmen wie Zugangsregelungen, Regeln für das Risikomanagement und die zweckgebundene Datenminimierung, aber auch Verfahrensregeln für den Fall einer unbeabsichtigten Identifizierung umfassen.

Außerhalb des Kontexts, in dem Daten als anonym gelten, ist die Wahrscheinlichkeit der Identifizierbarkeit weiterhin als variabler Faktor des Eingriffs in die Rechte und Freiheiten der betroffenen Personen nach allgemeinem Ermessen zu berücksichtigen. Sie kann Teil des Kontexts der Datenverarbeitung sein, der die Risiken der Verarbeitung bestimmt. Als Faktor beeinflusst sie auch die Frage, welche Vorkehrungen getroffen werden müssen, um die betroffenen Personen vor den Risiken der Verarbeitung im weiteren Sinne zu schützen. Daher spielt sie auch eine Rolle bei der Festlegung geeigneter technischer und organisatorischer Maßnahmen für die Datenverarbeitung, die der praktischen Umsetzung der Verhältnismäßigkeit dienen.

Indem sie helfen, den Kontext der Datenverarbeitung zu definieren, können sichere Datenräume dazu beitragen, die Risiken der Herstellung eines Personenbezugs und damit auch der Beeinträchtigung der Rechte und Freiheiten der Betroffenen zu minimieren. Ihre technische und rechtliche Ausgestaltung trägt darüber hinaus auch zur Umsetzung des Verhältnismäßigkeitsgrundsatzes im weiteren Verlauf der Verarbeitung bei. Die zugrunde liegende Verhältnismäßigkeitsabwägung kollidierender Interessen von Verarbeitern und Betroffenen wird im Gesundheitsbereich im Vergleich zu anderen Verarbeitungskontexten besonders dadurch beeinflusst, dass Verarbeitungsergebnisse oft nur personenbezogen Sinn ergeben und Betroffene ein stärkeres Interesse an der Verarbeitung sowie an der Rückbindung der Ergebnisse an ihre Person haben können. Sichere Datenräume könnten somit die Funktion von „Abwägungsräumen“ im Rahmen des Verhältnismäßigkeitsprinzips der DSGVO erfüllen, sowohl national als auch international. Die Schaffung eines günstigen regulatorischen Umfelds für die Verarbeitung von Gesundheitsdaten durch sichere Datenräume kann darüber hinaus als Grundlage für die Nutzung von Datenressourcen im öffentlichen Interesse zur Entwicklung der Gesundheitsversorgung und der forschungsbasierten translationalen Medizin unter Einhaltung klar definierter rechtlicher Voraussetzungen dienen.

## Footnotes

1 Im Duden wird „Risiko“ definiert als „möglicher negativer Ausgang bei einer Unternehmung, mit dem Nachteile, Verlust, Schäden verbunden sind; mit einem Vorhaben, Unternehmen o. Ä. verbundenes Wagnis“. Zum etymologischen Hintergrund siehe „Risiko“, bereitgestellt durch das Digitale Wörterbuch der deutschen Sprache, https://www.dwds.de/wb/Risiko. (Zugegriffen. 6. September 2022).

2 Für eine ausführliche Erläuterung dieser Definition im Unterschied zum Begriff der Gefahr s. Fehling, M (2019) Zur Bewertung von Entscheidungsfindungen. In: Fleischer B, Lauterbach R, Pawlik K (Hrsg.) Rationale Entscheidungen unter Unsicherheit, De Gruyter, Berlin, S. 124–129 [S. 125].

3 Verordnung (EU) 2016/679 des Europäischen Parlaments und des Rates vom 27. April 2016 zum Schutz natürlicher Personen bei der Verarbeitung personenbezogener Daten, zum freien Datenverkehr und zur Aufhebung der Richtlinie 95/46/EG (Datenschutz-Grundverordnung), OJ L 119, 04.05.2016, p. 1–88.

4 Die Liste wird als unsystematisch kritisiert, s. Schröder, M (2019) Der risikobasierte Ansatz in der DSGVO. ZD, S 503–506 [S. 504].

5 Konferenz der unabhängigen Datenschutzaufsichtsbehörden des Bundes und der Länder (2018) Kurzpapier Nr. 18, https://www.datenschutzkonferenz-online.de/media/kp/dsk_kpnr_18.pdf. (Zugegriffen: 6. September 2022) [S. 1].

6 S. Schröders Kritik an einer Überdehnung des Begriffs mit Verweis auf das Kurzpapier: Schröder, M (2019) Der risikobasierte Ansatz in der DSGVO. ZD, S 503–506 [S. 504].

7 Paal/Pauly/*Ernst*, 3. Aufl. 2021, DS-GVO Art. 1 [Rn. 8].

8 Calliess/Ruffert/*Kingreen*, 6. Aufl. 2022, EU-GRCharta Art. 52 [Rn. 65].

9 *Jarass*, in: Jarass GRCh Art. 52 [Rn. 36 mit Nachw. aus der Rspr.].

10 Europäischer Datenschutzausschuss (EDSA), Endorsement 1/2018 v. 25.05.2018 [S. 1] i. V. m. Art.-29-Datenschutzgruppe, Leitlinien zur Datenschutz-Folgenabschätzung (DSFA) und Beantwortung der Frage, ob eine Verarbeitung im Sinne der Verordnung 2016/679 „wahrscheinlich ein hohes Risiko mit sich bringt“ v. 04.10.2017, Dok. 17/DE, WP 248 Rev. 01 [S. 6 f.].

11 Gola DS-GVO/*Piltz*, 2. Aufl. 2018, DS-GVO Art. 24 [Rn. 27].

12 Taeger/Gabel/*Lang*, 4. Aufl. 2022, DS-GVO Art. 24 [Rn. 35].

13 Hoffmann-Riem W (2012) Eigenständigkeit der Verwaltung, in: Hoffmann-Riem W, Schmidt-Aßmann E, Voßkuhle A (Hrsg) Grundlagen des Verwaltungsrechts, Bd. 1, 2. Aufl. Beck, München, § 10 [Rn. 100].

14 Dieser Ansatz wird als „absoluter Ansatz zur Anonymität“ bezeichnet. Artikel-29-Datenschutzgruppe, Stellungnahme 05/2014 zu Anonymisierungstechniken, angenommen am 10.04.2014 [S. 7], s. allerdings [S. 19 f.]. Europäischer Datenschutzausschuss, Erklärung zur Verarbeitung personenbezogener Daten im Zusammenhang mit COVID-19, angenommen am 19.03.2020 [S. 2].

15 *Artikel-29-Datenschutzgruppe*, Stellungnahme 05/2014 zu Anonymisierungstechniken, angenommen am 10.04.2014 [S. 10].

16 *Artikel-29-Datenschutzgruppe*, Stellungnahme 05/2014 zu Anonymisierungstechniken, angenommen am 10.04.2014 [S. 16].

17 Dies gilt sowohl für den relativen als auch für den vermittelnden Ansatz. Zum relativen Ansatz s. Kühling/Buchner/*Klar/Kühling*, 3. Aufl. 2020, DS-GVO Art. 4 Nr. 1 [Rn. 26]. Zum vermittelnden Ansatz s. Taeger/Gabel/*Arning/Rothkegel*, 4. Aufl. 2022 DS-GVO Art. 4 [Rn. 35]. Der vermittelnde Ansatz wurde in der Rechtsprechung etabliert, s. EuGH, Rs. C‑582/14 (Breyer/Deutschland), EU:C:2016:779 [Rn. 31 ff., Rn. 43 ff.].

18 Simitis/Hornung/Spiecker gen. Döhmann/*Karg*, 1. Aufl. 2019, DSGVO Art. 4 Nr. 1 [Rn. 22].

19 Positionspapier für den Bundesbeauftragten für den Datenschutz und der Informationsfreiheit zur Anonymisierung unter der DSGVO mit besonderer Berücksichtigung der TK-Branche, 19.06.2020 [S. 4].

20 Gierschmann S (2021) Gestaltungsmöglichkeiten durch systematisches und risikobasiertes Vorgehen – Was ist schon anonym. ZD, S 482–486 [S. 483].

21 Für eine ausführlichere Darstellung der hier folgenden Beispiele vgl. Molnár-Gábor F, Beauvais MJS, Bernier A, Jimenez MPN, Recuero M, Knoppers BM (2022) Bridging the European Data Sharing Divide in Genomic Science. J Med Internet Res 2022;24(10):e37236. 10.2196/37236.

22 Bidgood WD Jr, Horii SC, Prior FW, & Van Syckle DE (1997) Understanding and using DICOM, the data interchange standard for biomedical imaging. Journal of the American Medical Informatics Association: JAMIA 4(3):199–212. 10.1136/jamia.1997.0040199.

23 Getz K, Farides-Mitchell J (2019) Assessing the adoption of clinical trial results summary disclosure to patients and the public. Expert review of clinical pharmacology 12(7):573–578. 10.1080/17512433.2019.1615441.

24 Wilkinson K, Green C, Nowicki D, & Von Schindler C (2020) Less than five is less than ideal: replacing the „less than 5 cell size“ rule with a risk-based data disclosure protocol in a public health setting. Canadian journal of public health = Revue canadienne de sante publique 111(5):761–765. 10.17269/s41997-020-00303-8.

25 Zu den Unterschieden unter den EU Mitgliedstaaten vor der unionsrechtlichen Harmonisierung, s. Polonetsky J, Tene O, Finsch K (2016) Shades of Grey, Seeing the full spectrum of technical data de-identification. Santa Clara Law Review 56(3):594–629 [S. 603].

26 Communication from the Commission to the European Parliament, the Council, the European Economic and Social Committee and the Committee of the Regions. A European strategy for data. Released on 19 February 2020 [S. 9].

27 Kühling/Buchner/Klar/*Kühling*, 3. Aufl. 2020, DS-GVO Art. 4 Nr. 5 [Rn. 11].

28 EuGH, Rs. C-582/14 (Breyer/Deutschland), EU:C:2016:779 [Rn. 31 ff.], mAnm *Kühling/Klar* ZD 2017, 24; wie hier wohl auch Roßnagel A (2018) Pseudonymisierung personenbezogener Daten. Ein zentrales Instrument im Datenschutz nach der DS-GVO. ZD, S. 243–247 [S. 245].

29 BeckOK DatenschutzR/*Schild*, 41. Ed. 01.08.2022, DS-GVO Art. 4 [Rn. 74–76].

30 Siehe bspw. § 13 Abs. 3 LDSG-BW.

31 Paal/Pauly/*Ernst*, 3. Aufl. 2021, DS-GVO Art. 4 [Rn. 40–47].

32 BeckOK DatenschutzR/*Schild*, 41. Ed. 01.08.2022, DS-GVO Art. 4 [Rn. 78].

33 Roßnagel A (2021) Datenlöschung und Anonymisierung. ZD, S 188–192 [S. 191 f.].

34 EuGH, Rs. C‑582/14 (Breyer/Deutschland), EU:C:2016:779 [Rn. 43 ff.].

35 Vgl. die Erläuterung der Unterschiede zwischen explicitly personal, potentially identifiable, key-coded, pseudonymous, de-identified, und anonymous data: Hintze, M (2017) Viewing the GDPR through a De-Identification Lens: A Tool for Compliance, Clarification, and Consistency. International Data Protection Law 8(1):86–101. 10.1093/idpl/ipx020. Polonetsky, J, Tene, O, Finsch, K (2016) Shades of Grey, Seeing the full spectrum of technical data de-identification. Santa Clara Law Review 56(3):594–629 [S. 603, S. 609 ff.].

36 Roßnagel A (2019) Datenschutz in der Forschung. ZD, S 157–164 [S. 162].

37 Polonetsky J, Tene O, Finsch K (2016) Shades of Grey, Seeing the full spectrum of technical data de-identification. Santa Clara Law Review 56(3):594–629 [S. 603, S. 605].

38 PHG Foundation (2020) The GDPR and genomic data – the impact of the GDPR and DPA 2018 on genomic healthcare and research [S. 35 ff.].

39 EuGH, Rs. C‑582/14 (Breyer/Deutschland), EU:C:2016:779 [Rn. 43 ff.].

40 Taeger/Gabel/*Arning/Rothkegel*, 4. Aufl. 2022, DS-GVO Art. 4 [Rn. 31].

41 Laue P (2019) § 1 Einführung. In: Laue P, Kremer S (Hrsg) Das neue Datenschutzrecht in der betrieblichen Praxis, 2. Aufl. Nomos, Baden-Baden [Rn. 16].

42 Mourby M, Mackey E, Elliot M, et al. (2018) Are ‚pseudonymised‘ data always personal data? Implications of the GDPR for administrative data research in the UK. Computer Law & Security Review 34(2):222–233. 10.1016/j.clsr.2018.01.002 [S. 228].

43 Roßnagel A (2021) Datenlöschung und Anonymisierung. ZD, S 188–192 [S. 189].

44 Paal/Pauly/*Martini*, 3. Aufl. 2021, DS-GVO Art. 32 [Rn. 25].

45 Gola/*Piltz*, 2. Aufl. 2018, DS-GVO Art. 32 [Rn. 22].

46 Alt U (2020) Datensicherheit, Datenschutz und Technik – ein risikoorientierter Ansatz. DS, S 169–172 [S. 169].

47 Veil W (2015) DS-GVO: Risikobasierter Ansatz statt rigides Verbotsprinzip – Eine erste Bestandsaufnahme. ZD, S 347–353 [S. 352].

48 Gola DS-GVO/*Schulz*, 2. Aufl. 2018, DS-GVO Art. 6 [Rn. 204].

49 Nicht zuletzt ist auch die Interessenabwägung nach Art. 6 Abs. 1 lit. f DSGVO hervorzuheben, die als Rechtsgrundlage ebenfalls über die Zulässigkeit der Datenverarbeitung entscheidet. Durch diese Rechtsgrundlage schreibt der Unionsgesetzgeber eine Abwägung der Interessen des Verarbeiters und der betroffenen Person und damit unmittelbar die Erfassung der Risiken für die Betroffenen durch die Verarbeitung vor. In die Interessenabwägung sind auch die Erwartungen des Betroffenen einzubeziehen.

50 Kühling/Buchner/*Buchner*, 3. Aufl. 2020, DS-GVO Art. 1 [Rn. 16].

51 Zur Kontextabhängigkeit der Schutzbedürftigkeit: Paal/Pauly/*Frenzel*, 3. Aufl. 2021, DS-GVO Art. 9 [Rn. 6].

52 Gola DS-GVO/*Piltz*, 2. Aufl. 2018, DS-GVO Art. 24 [Rn. 19].

53 Dies ist bspw. der Fall bei der Pflicht zur Benennung eines Vertreters gem. Art. 27 Abs. 2 lit. a DSGVO, für die Information der Aufsicht gem. Art. 33 Abs. 1 DSGVO und für die Benachrichtigung der betroffenen Person gem. Art. 34 Abs. 1 DSGVO.

54 Taeger/Gabel/*Lang*, 4. Aufl. 2022, DS-GVO Art. 24 [Rn. 32].

55 Artikel-29-Datenschutzgruppe, Statement on the role of a risk-based approach in data protection legal frameworks (14/EN WP 218), angenommen am 30.05.2014 [S. 2 f.].

56 Gola DS-GVO/*Piltz*, 2. Aufl. 2018, DS-GVO Art. 24 [Rn. 19].

57 Sydow/*Mantz*, 2. Aufl. 2018, DS-GVO Art. 25 [Rn. 21].

58 Paal/Pauly/*Martini*, DS-GVO Art. 32 [Rn. 48].

59 BeckOK DatenschutzR/*Paulus*, 40. Ed. 01.11.2021, DS-GVO Art. 32 [Rn. 10].

60 Kühling/Buchner/*Jandt*, 3. Aufl. 2020, DS-GVO Art. 32 [Rn. 7].

61 BeckOK DatenschutzR/*Paulus*, 40. Ed. 01.11.2021, DS-GVO Art. 32 [Rn. 8].

62 Helfrich N, Forgó M, Schneider J (2019) Kapitel 5 Grundsätze der datenschutzrechtlichen Prüfung. In: Helfrich M, Forgó N, Schneider J (Hrsg) Betrieblicher Datenschutz, 3. Aufl. C.H. Beck, München [Rn. 98].

63 Ehmann/Selmayr/*Hladjk*, DS-GVO Art. 32 [Rn. 11].

64 BeckOK DatenschutzR/*Paulus*, 40. Ed. 01.11.2021, DS-GVO Art. 32 [Rn. 7].

65 Kühling/Buchner/*Jandt*, 3. Aufl. 2020, DS-GVO Art. 32 [Rn. 36].

66 Wybitul T, Ströbel L, Ruess M (2017) Übermittlung personenbezogener Daten in Drittländer. ZD, S 503–509 [S. 504].

67 EuGH, Rs. C‑362/14 (Schrems/Data Protection Commission), EU:C:2015:650 [Rn. 73].

68 Kuner Ch (2017) Reality and Illusion in EU Data Transfer Regulation Post Schrems. GLJ 18:881.

69 Simitis/Hornung/Spiecker gen. Döhmann/*Schantz*, 1. Aufl. 2019, DS-GVO Art. 45 [Rn. 6].

70 EuGH, Rs. C‑293/12 und C‑594/12 (Digital Rights Ireland Limited/Minister for Communications, Marine, Natural Resources/Kärntner Landesregierung), EU:C:2014:238 [Rn. 45 ff.].

71 EuGH, Schlussanträge des Generalanwalts Paolo Mengozzi, Gutachten 1/15 (Gutachtenantrag des Europäischen Parlaments), EU:C:2016:656 [Rn. 195 ff.].

72 Vgl. Art. 45 Abs. 1 DSGVO und Art. 46 Abs. 1 DSGVO.

73 Europäischer Datenschutzausschuss, Empfehlungen 01/2020 zu Maßnahmen zur Ergänzung von Übermittlungstools zur Gewährleistung des unionsrechtlichen Schutzniveaus für personenbezogene Daten, angenommen am 10.11.2020 [S. 3].

74 BeckOK DatenschutzR/*Lange/Filip*, 40. Ed. 01.11.2021, DS-GVO Art. 46 [Rn. 12].

75 Siehe die weiterführenden Angaben der *Europäischen Kommission*: https://ec.europa.eu/info/law/law-topic/data-protection/international-dimension-data-protection/adequacy-decisions_de. (Zugegriffen: 6. September 2022).

76 Kuner Ch (2020) The Schrems II Judgment of the Court of Justice and the Future of Data Transfer Regulation. European Law Blog of 17 July 2020. https://europeanlawblog.eu/2020/07/17/the-schrems-ii-judgment-of-the-court-of-justice-and-the-future-of-data-transfer-regulation/. (Zugegriffen: 6. September 2022).

77 Weiterführend s. Kühling/Buchner/*Hartung*, 3. Aufl. 2020, DS-GVO Art. 4 Nr. 7 [Rn. 13 f.].

78 Europäischer Datenschutzausschuss, Guidelines 07/2020 on the concepts of controller and processor in the GDPR, Version 2.0., 07.07.2021 [Rn. 51].

79 Konferenz der unabhängigen Datenschutzbehörden des Bundes und der Länder (2018) Kurzpapier Nr. 16. https://www.datenschutzkonferenz-online.de/media/kp/dsk_kpnr_16.pdf. (Zugegriffen: 16. März 2021) [S. 1].

80 BeckOK DatenschutzR/*Spoerr*, 40. Ed. 01.05.2022, DS-GVO Art. 26 [Rn. 1–81].

81 Zu bestehenden Dispositionsmöglichkeiten s. Folkerts E (2022) Gemeinsame Verantwortlichkeit: Grenzen der Aufteilung datenschutzrechtlicher Verpflichtungen. ZD, S 201–206.

82 Ehmann/Selmayr/*Bertermann*, 2. Aufl. 2018, DS-GVO Art. 26 [Rn. 16].

83 Kühling/Buchner/*Bergt*, 3. Aufl. 2020, DS-GVO Art. 82 [Rn. 57 f.].

84 Instruktiv dazu: BeckOK DatenschutzR/*Spoerr*, 40. Ed. 01.05.2022, DS-GVO Art. 26 [Rn. 18 ff.].

85 Jauer ML, Deserno TM (2020) Data Provenance Standards and Recommendations for FAIR Data. Studies in health technology and informatics 270:1237–1238. 10.3233/SHTI200380.

86 Cheah PY, Piasecki J (2020) Data Access Committees. BMC medical ethics 21(1):12. Shabani M, Dove ES, Murtagh M, Knoppers BM, & Borry P (2017) Oversight of Genomic Data Sharing: What Roles for Ethics and Data Access Committees?. Biopreservation and biobanking, 15(5):469–474.

87 Sheller MJ, Edwards B, Reina GA, et al. (2020) Federated learning in medicine: facilitating multi-institutional collaborations without sharing patient data. Sci. Rep. 10:1–12.

88 Froelicher D, Troncoso-Pastoriza JR, Raisaro JL, et al. (2021) Truly privacy-preserving federated analytics for precision medicine with multiparty homomorphic encryption. Nat Commun. Oct 11;12(1):5910.

89 Bernier, A, Molnár-Gábor, F, Knoppers, BM (2022) The international data governance landscape. Journal of law and the biosciences 9(1): lsac005. 10.1093/jlb/lsac005.

90 Thorogood A, Rehm HL, Goodhand P, et al. (2021) International Federation of Genomic Medicine Database Using GA4GH Standards. Cell Geonomics 1(2):100032. Suver Ch, Thorogood A, Doerr M, Wilbanks J, Knoppers BM (2020) Bringing Code to Data: Do Not Forget Governance. J. Med. Internet Res. 22:e18087.

91 Froelicher D, Troncoso-Pastoriza JR, Raisaro JL, et al. (2021) Truly privacy-preserving federated analytics for precision medicine with multiparty homomorphic encryption. Nat Commun. Oct 11;12(1):5910.

92 Molnár-Gábor F, Beauvais MJS, Bernier A, Jimenez MPN, Recuero M, Knoppers BM (2022) Bridging the European Data Sharing Divide in Genomic Science. J Med Internet Res 2022;24(10):e37236. 10.2196/37236.

93 Europäischer Datenschutzausschuss, Guidelines 05/2021 on the Interplay between the application of Article 3 and the provisions on international transfers as per Chapter V of the GDPR, angenommen am 18.11.2021.

94 EuGH, Rs. C‑101/01 (Lindqvist), EU:C:2003:596 [Rn. 53 ff.].

95 Molnár-Gábor F, Beauvais MJS, Bernier A, Jimenez MPN, Recuero M, Knoppers BM (2022) Bridging the European Data Sharing Divide in Genomic Science. J Med Internet Res 2022;24(10):e37236. 10.2196/37236.

96 Vorschlag für eine Verordnung des Europäischen Parlaments und des Rates über den europäischen Raum für Gesundheitsdaten, COM/2022/197 final.

97 Tschammler D, Uecker P (2022) Verordnungsentwurf zur Schaffung eines Europäischen Gesundheitsdatenraums – erster Überblick mit Fokus auf die Nutzung von Gesundheitsdaten für Forschungs- und Entwicklungsvorhaben. ZD-Aktuell 01265.

98 BeckOK DatenschutzR/*Schild*, 41. Ed. 01.08.2022, DS-GVO Art. 4.

99 ErwG 75 DSGVO.

